# Histone methylation in pre-cancerous liver diseases and hepatocellular carcinoma: recent overview

**DOI:** 10.1007/s12094-023-03078-9

**Published:** 2023-01-17

**Authors:** Evelina Charidemou, Costas Koufaris, Maria Louca, Antonis Kirmizis, Teresa Rubio-Tomás

**Affiliations:** 1 Department of Biological Sciences, University of Cyprus, 2109 Nicosia, Cyprus; 2 School of Medicine, University of Crete, 70013 Herakleion, Greece

**Keywords:** Histone methyltransferases, Histone demethylases, Hepatocellular carcinoma, Non-alcoholic fatty liver disease, Viral hepatitis, Environmental carcinogens

## Abstract

Hepatocellular carcinoma (HCC) is the prevalent form of liver cancer in adults and the fourth most common cause of cancer-related death worldwide. HCC predominantly arises in the context of cirrhosis as a result of chronic liver disease, injury and inflammation. Full-blown HCC has poor prognosis because it is highly aggressive and resistant to therapy. Consequently, interventions that can prevent or restrain HCC emergence from pre-cancerous diseased liver are a desirable strategy. Histone methylation is a dynamic, reversible epigenetic modification involving the addition or removal of methyl groups from lysine, arginine or glutamine residues. Aberrant activity of histone methylation writers, erases and readers has been implicated in several cancer types, including HCC. In this review, we provide an overview of research on the role of histone methylation in pre-cancerous and cancerous HCC published over the last 5 years. In particular, we present the evidence linking environmental factors such as diet, viral infections and carcinogenic agents with dysregulation of histone methylation during liver cancer progression with the aim to highlight future therapeutic possibilities.

## Introduction

Hepatocellular carcinoma (HCC) is the most common form of primary liver cancer, accounting for 70–85% of liver cancer cases worldwide [1, 2]. The survival of HCC patients is very poor, with only 5% of patients surviving more than 5 years due to the asymptomatic development and progression of HCC at an early stage and aggressive cancer cell metastatic behaviour at a later stage. HCC emerges predominantly in a setting of chronic liver disease background, with the underlying aetiology, however, varying considerably between regions and sociodemographic index [3]. The largest contribution to HCC globally relates to viral infections, with hepatitis B virus (HBV) infection and hepatitis C virus (HCV), estimated to, respectively, cause 43 and 18% of all liver cancer deaths globally in 2016. The greatest prevalence of these viral infections and their contributions to HCC occurs in East Asia. Alcohol consumption is another important cause of HCC, associated with around 15% of liver cancer deaths globally in 2016, with the contribution being considerably higher in several Western countries. An increasing aetiology of HCC, especially in Western countries, is non-alcoholic fatty liver disease (NAFLD) [4]. Chronic NAFLD can lead to two pathological forms: steatosis and non-alcoholic steatohepatitis (NASH). Isolated hepatic steatosis is a benign condition characterised by fat accumulation; however, if this condition progresses, it is associated with inflammation and eventually causes NASH. Persistent inflammation results in the formation of scar tissue (fibrosis) leading to cirrhosis [5]. NAFLD-associated HCC is mostly preceded by cirrhosis but can also directly develop from NASH [6]. Although vaccinations and the advent of effective anti-HCV therapies have been successful in decreasing the incidence of viral-associated HCC, there are increasing rates of liver cancer due to increased risk factors such as diabetes and obesity that can cause NAFLD [3, 7].

Despite the development and identification of drugs for HCC treatment such as the kinase inhibitors, sorafenib (Nexavar) and lenvatinib, which are used as frontline treatments, as well as other second-line treatments (i.e. regorafenib and cabozantinib), the overall survival of HCC patients has not improved [8]. The development of new, more effective therapeutic drugs against HCC will most probably emerge through a deeper understanding of the molecular and cellular events that promote hepatocarcinogenesis. One such promising avenue is the perturbation of the liver epigenetic landscape in pre-cancerous and cancerous liver by the action of aforementioned HCC risk factors. The term epigenetics refers to changes in chromatin states that are not attributed to alterations in DNA sequence. Epigenetic mechanisms underlie the unique ability of the liver among mammalian solid organs to coordinate and control its regenerative capacity and ability to adapt to a rapidly changing environment [9, 10]. One crucial epigenetic mechanism in normal and diseased liver that comprises the focus of this review is methylation of histones. In eukaryotic cells, each nucleosome consists of pairs of histone proteins H2A, H2B, H3 and H4 with DNA wrapped around them [11]. Methylation of histones occurs primarily at lysine or arginine residues through the activity of methyltransferase enzymes that use S-adenosyl methionine as the donor molecule. Acknowledged histone tail and globular residues known to carry methylation marks of molecular significance include H3K4, H3K27, H3K36, H3K79, H4K20, H3K23, H3K63 and H4K12 [12]. Lysine methyltransferases (KMTs) represent a large class with more than 100 known members. KMTs can mono, di or tri-methylate lysine residues, with enzymes differing in their sequence preference and ability to mediate all three levels of lysine methylation. For example, KMT6 also known as EZH2 is a component of the PCR2 complex that specifically mediates trimethylation of histone H3 lysine 27 (H3K27me3) histone mark while DOT1L generates H3K79me3 [12]. Methylated arginine can be found as monomethylated, asymmetrically dimethylated, or symmetrically dimethylated [13]. Protein arginine methyltransferases (PRMTs) are divided into three types based on their ability to deposit the distinct types of arginine methylations. Well-characterised histone arginine methylated residues include H2AR3, H4R3, H3R2, H3R8 and H3R26 [14–16].

The identification of demethylases has confirmed the dynamic nature of histone methylation marks. Removal of lysine methylation is mediated by demethylases that are classified into two classes according to their mode of action, nucleophilic catalysis and dependence on oxidative catalysis [17]. Arginine methylation was proposed to be removed through the action of the Jumonji domain-containing 6 (JMJ6) [18], but mainly methylated arginines can be converted to citrulline through the action of peptidyl arginine deiminase 4 (PADI4) [19].

“Readers” are the effector proteins that recognise histone marks depending on their neighbouring amino acid sequence and methylation state. These reader proteins contain one of a range of methyl-binding domains that bind to the histone mark and recruit transcriptional complexes or chromatin remodelling proteins to affect downstream processes [20].

Histone methylation marks are of interest due to their diverse effects on cell and organismal phenotypes. Histone methylation marks are associated with both increased (e.g. H3K4me3) or repressed (e.g. H3K9me3) transcription [21]. Since methylation carries a neutral charge and is a small molecule, it is not considered to act by affecting chromatin structure, but rather by influencing the recruitment and binding of effector molecules [22]. For example, the deposition of the H3K4 methylation mark by Set9 can block the association of the transcription repressor NuRD complex with the histone H3 tail [23].

As there have been hundreds of published studies on histone methylation and its involvement in pre-cancerous liver disease and HCC, we have focussed our attention in this review on important recent advances, covering the past 5 years. We first consider histone methylation responses to environmental risk factors in pre-cancerous liver, then in HCC proper, and we conclude with a brief summary of the current state of knowledge.

## Histone methylation in pre-cancerous chronic liver disease

### Gene–diet interaction in NAFLD

**Fig. 1:**
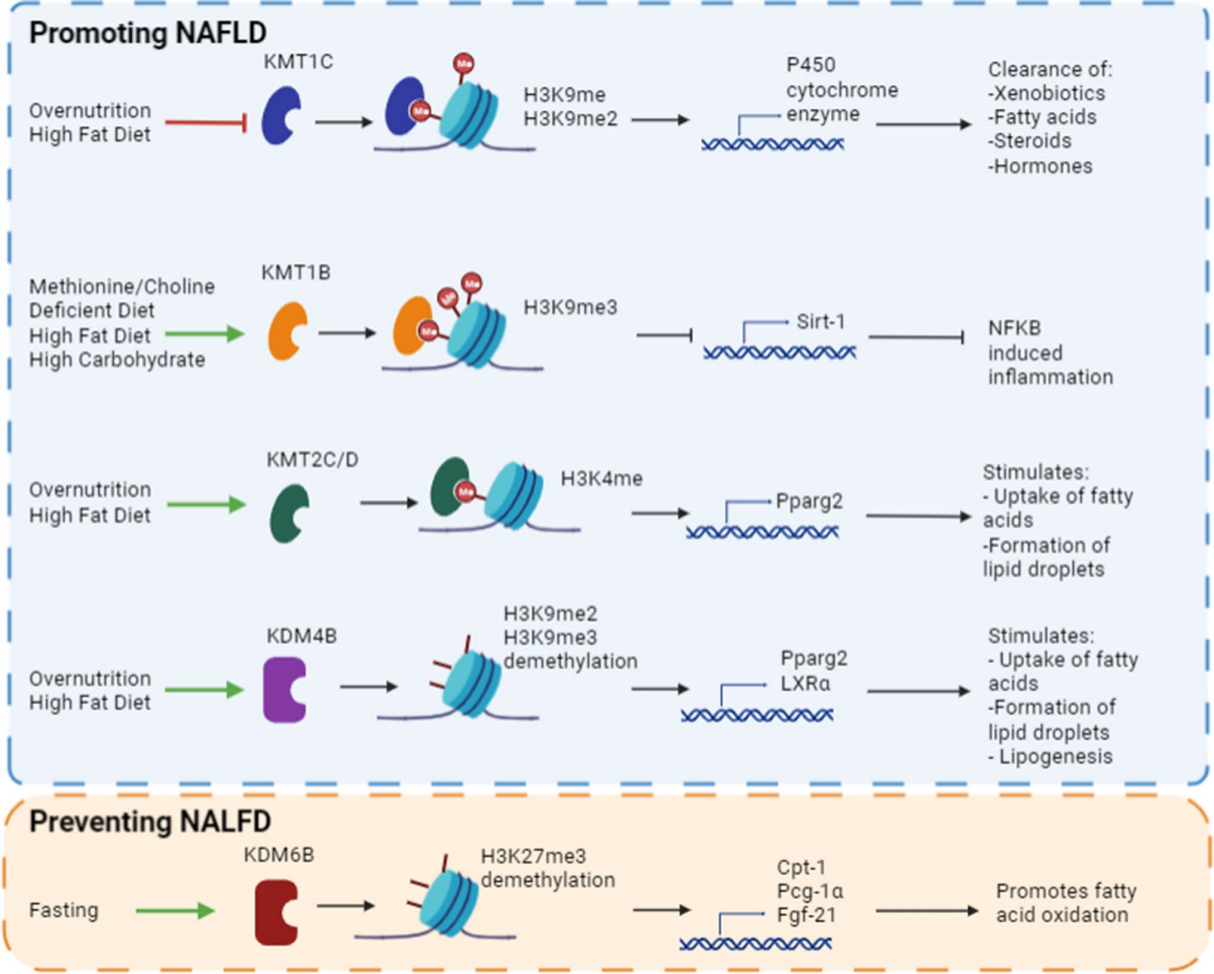
Dietary patterns that influence gene expression through histone methyltransferases and demethylases in NALFD progression. Top (blue) panel shows epigenetic modifiers that promote the progression of NAFLD, and bottom (orange) panel shows protective epigenetic modifiers that prevent the progression of NAFLD

In this section of the review, we will focus on the contribution of dietary patterns that influence gene expression through histone methyltransferases and demethylases in the progression of NAFLD (Fig. 1).

Histone methyltransferase KMT1C (also known as G9a) is responsible for the mono- and di-methylation of histone H3 at lysine 9 (H3K9). This epigenetic modifier is known to be downregulated by diet-induced obesity in animal models and was found to control the expression of cytochrome P450 that regulates the clearance of many compounds, including xenobiotics, fatty acids, steroids, and hormones [24]. In a recent study by Pande and colleagues (2020), it was shown that overexpression of the histone modifier KMT1C prevented the repression of nuclear receptors, androstane receptor, pregnane X receptor, and small heterodimer partner in a fatty acid-induced cellular model of steatosis. All of these are transcription factors that control the expression of the drug-metabolising P450 genes. Decreased P450 expression can lead to inefficient pharmacological effect of drugs or hepatotoxicity. This study suggested that H3K9 mono- and di- methylation should be considered as an epigenetic mechanism that controls P450 expression in early steatotic conditions and can, therefore, control the regulation of progression of fatty liver [24].

Besides mono- and di-methylation of H3K9 mediated by KMT1C, the suppressor of variegation 39 homolog 2 (KMT1B; also known as Suv39H2) catalyses histone H3 lysine 9 trimethylation and has also been implicated in the pathogenesis of NASH. In fact, it was shown that mice lacking KMT1B had a less severe form of high fat, high carbohydrates as well as a methionine/choline-deficient diet (MCD)-induced NASH [25, 26]. Pro-NASH stimuli increased the expression levels of KMT1B in liver in vitro and in vivo*,* while KMT1B deficiency allowed normal expression of Sirtuin (Sirt-1), which, in turn, inhibited the nuclear factor kappa B (NFkB)-dependent transcription of proinflammatory mediators [25]. In agreement, another study showed that H3K9 trimethylation by KMT1B repressed Sirt-1 expression in the liver, while KMT1B deficiency alleviated Sirt-1 expression in MCD-fed mice [26]. Both studies propose that histone methyltransferase KMT1B has a critical role in the pathogenesis of NASH as it controls diet-induced hepatic inflammation through Sirt-1 and NFkB expression.

Another critical epigenetic regulator of overnutrition-induced hepatic steatosis is the histone H3 lysine 4 methyltransferase mixed-lineage leukaemia 4 (KMT2D; also known as MLL4). In a recent study, KMT2D was demonstrated to direct overnutrition-induced steatosis in mice through the activation of the nuclear receptor peroxisome proliferator activated receptor γ isoform 2 (PPARγ2), the master adipogenic transcription factor that stimulates the uptake of fatty acids and the formation of lipid droplets, thereby promoting hepatic steatosis. Kim and colleagues (2016) showed that overnutrition activated the kinase ABL1, an effector for fat storage, and this in turn phosphorylated and activated PPARγ2. Thereafter, the PPARγ2 transcription factor recruited KMT2C/D complexes to a large fraction of genes including the *Pparg2* gene promoting fatty liver formation through H3K4 methylation [27].

In addition to methyltransferases, histone lysine demethylases have also been implicated in the progression of NALFD. Specifically, the α-ketoglutarate-dependent Jumonji C (JmjC) domain-containing demethylase KDM4B (also known as JMJD2B) has been reported to promote hepatic steatosis by regulating the adipogenic transcription factor, PPARγ2, and lipogenic transcription factor, LXRα [28, 29]. KDM4B is responsible for the removal of di- and tri-methylated H3K9 (H3K9me2/me3), catalysing their conversion back to the monomethylated state. It has previously been shown that KDM4B induced the expression of PPARγ2 by removing H3K9me2/me3 on the PPARγ2 promoter, and thereby activating adipogenesis in pre-adipocytes [29]. Moreover, the expression of PPARγ2 is increased in in vitro and in vivo steatotic models [28]. In fact, overexpression of KDM4B in HepG2 hepatocyte cell line increased the expression of PPARγ2 as well as its associated steatosis-related genes promoting fatty acid uptake and lipid droplet accumulation. The overexpression of KDM4B in HepG2 cell line and in high-fat diet-fed mice reduced the enrichment of H3K9me2 and H3K9me3 on the promoter of PPARγ2, thereby stimulating its expression as well as the expression of PPARγ2 steatotic target genes (fatty acid translocase, CD36; fatty acid binding protein 4, FABP4; perilipin 2, PLIN2; fat-specific protein 27/cell death-inducing DFFA-like effector C, CIDEC) that promote the progression of NAFLD [28].

Histone H3K9 demethylase, KDM4B, was recently reported to also contribute to hepatic steatosis through its control of the lipogenic transcription factor, ligand-activated liver X receptor α (LXRα) [30]. Overexpression of KDM4B in steatotic hepatocytes prompted its recruitment and that of LXRα to LXR response elements (LXRE) in the promoter region of LXRα-target genes, where there was reduced enrichment of H3K9me2 and H3K9me3 increasing the expression of LXRα-dependent lipogenic genes (such as fatty acids synthase, acetyl CoA carboxylase, and stearoyl-CoA desaturase 1). This induction of lipogenic genes induced the accumulation of triglycerides in liver cells. In agreement, overexpression of KDM4B in the liver of mice stimulated LXR-dependent lipogenesis and induced hepatic steatosis [30].

Taken together, the results of different studies indicate that KDM4B plays a critical role in PPARγ2-dependent fatty acid uptake and storage as well as LXR-mediated lipogenesis by removing the repressive histone marks, H3K9me2 and H3K9me3, on their corresponding promoters, contributing to the formation of liver steatosis.

Unlike KDM4B, Jumonji D3 (KDM6B/JMJD3), which catalyses the removal of methyl groups from histone H3K27me3, has been described to be protective against NALFD [31, 32]. Specifically, it has been reported that fasting promotes the interaction of the demethylase KDM6B with the transcription factor Sirt-1 and the nuclear receptor peroxisome proliferator activated receptor α (PPARα) in the liver to activate transcription of mitochondrial fatty acid β-oxidation genes, including Cpt1, Pgc-1α and Fgf21 through decreased levels of H3K27me3. In fact, the lipid-lowering effects mediated by expression of SIRT1 in obese mice fed with high-fat diet are dependent on hepatic KDM6B and vice versa [32].

In addition to its lipid-lowering effect, KDM6B has also been implicated in hepatic autophagy, another biological process that is dysregulated in NAFLD patients. A recent study showed that nutrient deprivation stimulated the hepatokine fibroblast growth factor-21 (FGF21), which in turn activated the demethylase KDM6B to interact with the transcription of PPARα to also activate autophagy-related genes, via demethylation of histone H3K27me3. In obese/steatotic mice, FGF21 administration ameliorated defective autophagy in a KDM6B-dependent manner [31].

Taken together, these findings demonstrate that demethylase KDM6B epigenetically links nutrient starvation to the regulation of autophagy and breakdown of lipids through β-oxidation to protect against the progression of steatosis as these processes are dysregulated in NAFLD.

### Hepatitis B virus (HBV) infections

**Fig. 2:**
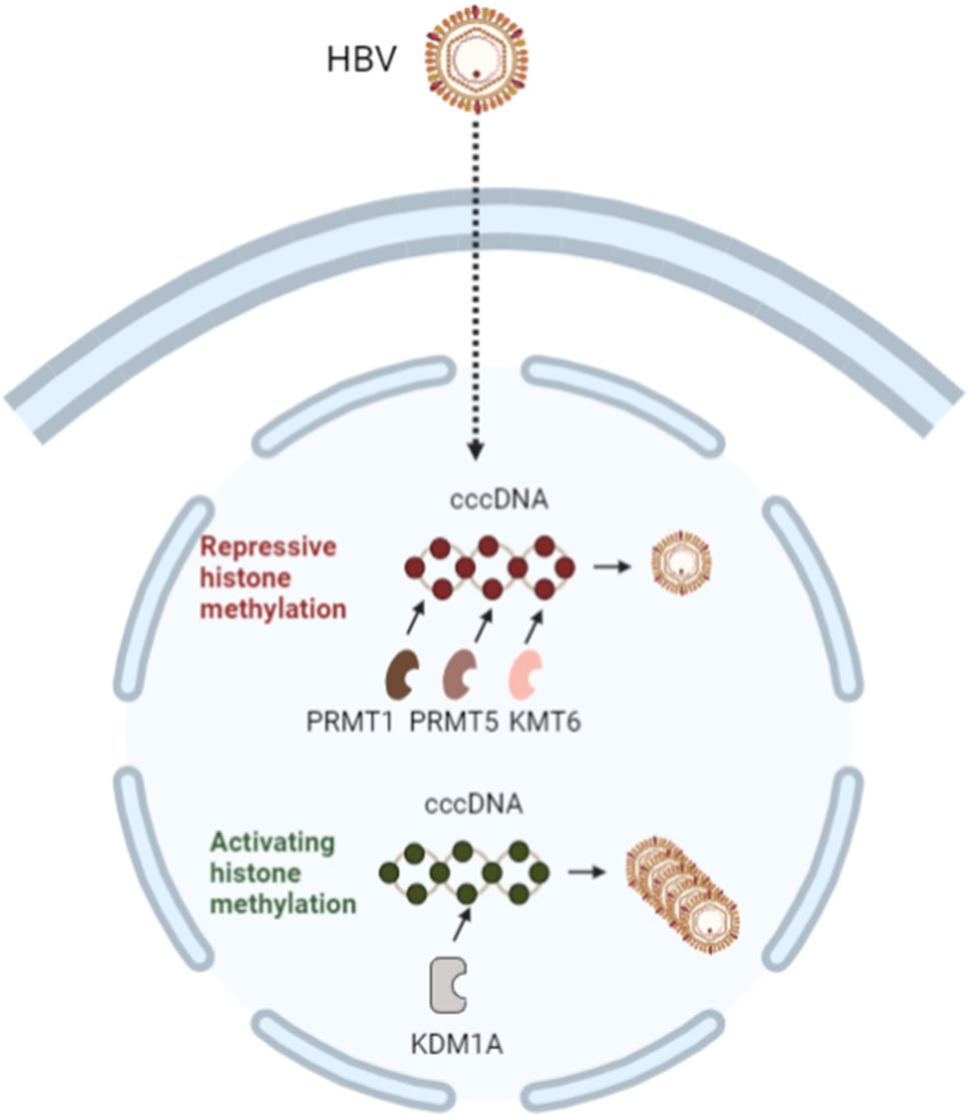
Regulation of cccDNA by histone methylation. HBV has a relaxed DNA genome that within the nucleus is transformed into cccDNA. The cccDNA persists within infected cells to act as template for production of viral particles. The rate of transcription of viral particles from the cccDNA is regulated among other factors by histone methylation and the activity of methyltransferases and demethylases

Once the HBV genomic DNA enters the nucleus of the host cell, it is converted into covalently closed circular DNA (cccDNA), that is crucial for chronic, persistent viral infection. This cccDNA organises into a minichromosome containing histone proteins H3 and H4, as well as non-histone proteins [33]. PTMs are distributed non-randomly across the cccDNA-associated histones, with high levels of modifications associated with activation (e.g. H3K4me3) and low levels of repressive marks (e.g. H3K27me3) [34], potentially playing a role in fine tuning the transcription of viral genes (Fig. 2).

The viral encoded proteins HBV X protein (HBx) and core protein (HBc) are known to physically interact with cccDNA [35], and there is evidence linking HBx activity to histone methylation of cccDNA. First, HBx recruits KDM1A (also known as LSD1) to cccDNA and the subsequent demethylation drives the transcription of viral genes and viral replication [36]. HBx also promotes viral gene transcription through its effects on deacetylase SIRT3 [37]. This deacetylase acts to remove H3K9ac and recruits methyltransferases to cccDNA, leading to increased H3K9 and decreased H3K4 methylation. These molecular changes lead to decreased transcription of viral genes in infected cells. HBx blocks both the transcription of SIRT3 and its recruitment to cccDNA and also directly binds the promoter and activates the transcription of the long noncoding RNA (lncRNA) DLEU2. Subsequently, HBx is physically associated with KMT6 and acts to displace KMT6 from cccDNA, allowing viral gene transcription [38]. Arginine methylation of cccDNA has also been implicated in the regulation of viral gene transcription. The arginine methyltransferase PRMT1 was shown to interact directly with HBx, with this interaction repressing the ability of the enzyme to methylate the cccDNA histone proteins. In the absence of HBx, PRMT1 methylation reduced the transcription of viral proteins [39]. Zhang and colleagues (2017) used an RNAi screen for selected histone methyltransferases and demethylases to determine their role in the regulation of transcription from cccDNA [40]. With this method, they found that PRMT5 silences transcription by depositing H4R3me2 marks on the cccDNA. In addition, in the same study, the authors reported that PRMT5 also repressed viral replication independent of its enzymatic activity by affecting pregenomic RNA encapsidation. HBx also reduced PRMT5 activity indirectly by driving the ubiquitination and degradation of the WDR77 protein that enhances the activity of the methyltransferase [41]. Recently, it was also demonstrated that isoforms of histone modifying enzymes can also have distinct effects on the epigenetics of cccDNA regulation, increasing the complexity of this mechanism. HBV infection increases the expression of multiple SIRT2 isoforms, which are mainly cytoplasmic. However, these researchers found that the SIRT2.5 isoform, which is catalytically inactive, is mainly nuclear. In the nucleus, the SIRT2.5 isoform recruits multiple HMTs to cccDNA, promoting the deposition of repressive histone methylation marks and reducing the expression of viral genes [42].

Overall, understanding the mechanisms controlling viral gene transcription from cccDNA could lead to new strategies for eliminating the virus from infected cells. HBV infection has shown to have strong effects on genomic histone methylation of infected liver cells in cell models and patient samples. Examination of HBV-infected cells by chromatin immunoprecipitation sequencing (ChIP-Seq) in the HepG2 hepatocyte cell line revealed widespread changes in H3K4me3 and HeK27me3 [43]. In a subsequent study, the authors reported that global increases in H3K4me3 occur post-transcriptionally through HBx blocking ubiquitination of the WD repeat domain 5 protein (WDR5), a subunit of complexes that deposit this modification [44]. In a follow-up study, the same group reported WDR5-driven deregulation of H3K4 methylation of the *ALKBH5* promoter. Increased levels of the ALKBH5 enzyme then catalyse m6A demethylation of HBx mRNA, stabilising and enhancing the viral protein (Q. S et al. 2021).

Recent studies have reported that beyond its effects on WDR5, HBV can affect histone methylation through alterations in lncRNA. One study investigating the impact of HBV infection on lncRNA in hepatic LO2 cells concluded that one of the affected lncRNAs, namely UCA1, recruits KMT6 to repress p27Kip1/CDK2 signalling [46]. A second group reported that another lncRNA, PVT1a, promotes invasiveness of HBV-infected cells by blocking histone methyltransferase KMT6-mediated repression of MYC target genes [47].

HBV can also decrease the effect of demethylases. Overexpression of HBx in hepatoma cells was shown to decrease KDM4B demethylase and upregulate H3K9me3. Immunohistochemistry revealed a strong negative correlation between HBx-positive HCC samples and KDM4B. Increased H3K9me3 was also observed on the promoter of p16, being potentially involved in the suppression of this tumour suppressor in HCC [48]. Thus, it is clear that HBV induces strong regulation of host genes through epigenetic mechanisms.

### Hepatitis C (HCV) infections

Increasing attention has recently been given to the extensive epigenetic effects of HCV on infected liver cells. Wijetunga and colleagues (2016) compared the transcriptomic and epigenetic state of HCV-infected pre-neoplastic and cancer tissue, as well as normal liver. Besides extensive effects on DNA methylation, the authors also found increased H3K27me3 enhancer methylation of PCR2-KMT6 target genes involved in stem cell development. Targeting KMT6-mediated histone methylation KMT6 with the GSK343 inhibitor in HepG2 repressed cancer cell growth, suggesting that this histone methylation mark is involved in regulation of genes important for the survival of liver cancer cells [49].

Independent groups have recently described that epigenetic effects of HCV infection, including histone methylation, are maintained even after clearance of viral infection. The first study examined the epigenomic effects of HCV infection on Huh7.5 hepatoma cells. No differences were observed for the H3K27me3 mark, while significant changes were observed for H3K4me3 within genes and regulatory regions which correlated with gene expression changes in these cells. Interestingly, the majority of the affected histone marks were retained after pharmacological removal of the infecting virus [50]. An independent study by a second group examined the epigenomic effects of HCV infection on two isogenic hepatocyte-derived models [51]. After long-term culture, cells were treated with either interferon-α or a direct-acting antiviral (telaprevir). They reported an increase in H3K4me1 (mark of enhancers) and decrease of H3K27me3. Again, altered histone methylation was observed after clearance of the viral infection, suggesting persistent epigenetic effects.

### Carcinogenic factors

These factors include occupational (e.g. heavy metals, vinyl chloride), lifestyle (e.g. alcohol, smoking) and medicinal agents (e.g. oral contraceptives, anabolic steroids), which act by causing DNA mutations and/or chronic inflammation leading to chronic liver injury [52].

There is evidence supporting the contention that exposure to heavy metals can drive carcinogenesis, including in the liver [53]. The mechanisms by which metals can drive carcinogenesis are still being debated, with evidence supporting that epigenetic disruption is also occurring and could be implicated. Cadmium originating from the diet accumulates in the liver of individuals with low exposure to this metal. Cadmium treatment of the HepG2 cell line for 24 h was sufficient to induce widespread transcriptomic and epigenetic changes. Examination of histone marks revealed a global reduction in H3K4 and H3K9 methylation, that was maintained at even 72 h after discontinuation of the treatment. However, this study did not examine whether the observed histone methylation changes related to altered gene expression [54]. Tungsten is a naturally occurring element, with the highest human exposures associated with occupational sources. Laulicht-Glick and colleagues (2017) investigated the effect of Tungsten exposure on global histone methylation. Exposure of mice to the metal added to their drinking water for 2 weeks caused increases in the levels for H3K9me2 and H3K4me3. Possibly this occurs through the degradation of two histone demethylase dioxygenases, KDM5A (a.k.a. JARID1A) and KDM3A (a.k.a. JMJD1A) (Glick et al. 2017). Arsenic is a natural, non-metal element that is widely distributed in the environment. Arsenic poisoning can cause liver damage and eventually progress to cirrhosis and HCC. As arsenic is a weak mutagen, its mode of action is considered to be primarily epigenetic. A number of recent studies have offered support to the possibility that altered histone methylation is an important event in arsenic-associated liver carcinogenesis. Han and colleagues (2021) investigated the role of SET7/9 H3K4 methyltransferases and LSD1 demethyltransferase in arsenic-induced apoptosis in liver cells [56]. Arsenic treatment of LO2 normal hepatocytes caused induction of H3K4me1 and SET7/9, while LSD1 was decreased. Importantly, manipulation of these epigenetic enzymes could alleviate arsenic-induced apoptosis. Finally, increased H3K4 methylation was observed in the promoter region of endoplasmic reticulum stress-related proteins GRP78 and CHOP. Zhang and colleagues (2016) investigated the effect of arsenic on the H3K9 methylation mark [57]. Treatment in the liver cells caused an increase of H3K9 globally and within the promoter of pyruvate dehydrogenase kinase isoenzyme 4 (*PDK4*), a key mitochondrial enzyme. Moreover, arsenic could block the increase in *PDK4* caused by KMT1C inhibitor BRD4770. Another recent study linked arsenic-induced deregulation of H3K9me2 with ineffective DNA repair in normal liver cells [58]. In this study, it was reported that arsenic exposure in liver cells caused the upregulation of the histone demethylase KDM3A and a global decrease in the levels of H3K9me2. At the same time, increased H3K9me2 was observed in the promoters of genes involved in DNA repair, which resulted in their decreased expression. Tryndyak and colleagues (2020) [59] examined the effects of exposure to low levels of sodium arsenite of the human non-carcinogenic HepaRG cell line. It was found that this treatment had prominent effects globally on both DNA and histone methylation. For histone methylation, a big decrease of H3K36me3 and a slight increase of histone H4K20me3 marks were observed. ChIP-seq for H3K36me3 found predominantly an arsenic-induced decrease predominantly in intronic and intergenic regions. The authors suggested that the observed decrease in this histone mark could be associated with genomic instability.

### Dysregulated histone methylation in HCC

**Fig. 3:**
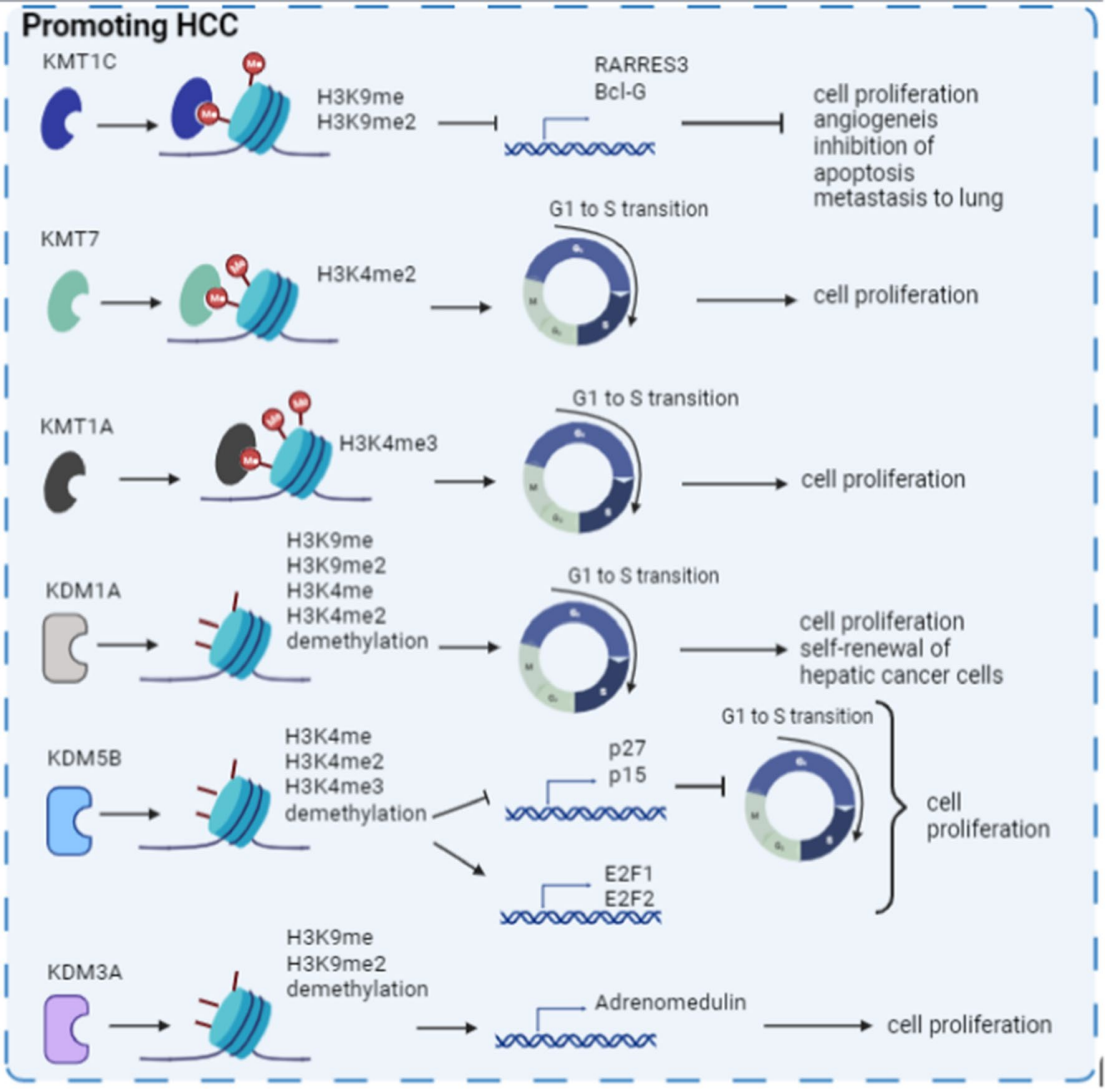
Examples of methyltransferases and demethylases deregulated in HCC and their functions in disease progression

Altered activity of both methyltransferases and demethylases is prominent in HCC proper. Recent data regarding histone methylation and HCC will be summarised in this section (Fig. 3).

Mounting evidence shows that HCC patients with high KMT1C expression and high levels of H3K9 di-methylation have worse survival outcomes due to KMT1C-dependent regulation of cell proliferation, angiogenesis, and anchorage of independent growth [60–64]. Wei and colleagues (2017) showed that KMT1C is upregulated in HCC and negatively regulates the tumour suppressor phospholipase gene, PARPES3, by H3K9 methylation, resulting in uncontrolled cell proliferation [64]. In addition, it has been shown that KMT1C silencing, through different RNAi approaches and CRISPR/Cas9 knockdown in subcutaneous xenograft model, inhibits tumour growth as well as lung metastasis. The same results were obtained using a chemical inhibitor for KMT1C methyltransferase. Interestingly, they also observed that KMT1C expression was gradually increased along the multiple stages of liver carcinogenesis indicating that KMT1C has a crucial role not only in the development of HCC but also in HCC progression and malignancy. Moreover, Yuan and colleagues (2021) showed that KMT1C promotes HCC cell growth in vitro using clonogenic cell survival assays and in vivo by constructing an orthotopic mouse model. Of note was the demonstration that KMT1C downregulates the expression of Bcl-G (a proapoptotic gene) and inhibits apoptosis through the p53 tumour suppressor gene leading to HCC development [65].

KMT7 (also known as SETD7) was shown to be overexpressed in HCC tumour tissues compared to adjacent non-tumoural liver tissues, and it was shown to be positively correlated with tumour size and poor tumour differentiation leading to poor prognosis in HCC patients [66]. Briefly, researchers showed that on KMT7 knockdown the population of HCC cells increased in the G1 phase showing that the KMT7 methyltransferase promotes the transition of HCC cells from G1 to S phase.

Chiba and colleagues (2015) reported that silencing this methyltransferase reduced HCC cell growth and arrested cells in the G0/G1 phase. Moreover, pharmacological inhibition of KMT1A (also known as SUV39H1) with chaetocin increased apoptosis of HCC cells in vitro and in xenograft subcutaneous tumours [67].

KDM1A has been shown to demethylate non-histone substrates such as p53, DNMT1, STAT3 and E2F1 [68]. In addition, KDM1A is overexpressed in HCC patient samples and that its expression is positively correlated with poor overall patient survival [69, 70]. Moreover, it was described that this demethylase is responsible for the maintenance of self-renewal in liver cancer stem cells [71]. Wu and colleagues (2019) revealed that, with the appropriate siRNA or by the suppression of its function with the specific inhibitor SP2509, KDM1A knockdown increased the efficiency of regorafenib, a second-line agent that targets and inhibits tyrosine kinase receptors [72] in HCC cells and its cytotoxic and apoptotic effects suppressing the proliferation of HCC cells [69]. Kim and colleagues (2019) found that KDM1A knockdown using the CRISPR/Cas9 system caused increased deposition of mono- and di-methylation of H3K4 and H3K9, and these histone modifications led to G1/S cell cycle arrest, impairing HCC colony formation [70]. In addition, it was shown that the silencing of KDM1A impairs the Wnt/b-catening signalling pathway and sorafenib resistance [73].

The KDM5B (JAR1D1B) is a jmjc domain-containing histone demethylase that belongs to the KDM5 family. Expression of this demethylase was studied using the GEO profiles database, and it was shown to be upregulated in HCC tissues compared to adjacent normal tissues. In addition, Kaplan–Meier survival analysis revealed that its expression is positively correlated with poor prognosis, with immunohistochemical analysis in 105 HCC clinical tissue samples validating these results [74, 75]. Patients with HCC were divided into those with high or low levels of KDM5B expression. Interestingly, patients with higher KDM5B expression after surgery had a poorer prognosis compared to patients with lower levels of this demethylase [75]. Moreover, silencing of KDM5B in vitro and in vivo impaired HCC cell proliferation and colony formation by blocking G1/S transition through upregulation of CDK1 p15 and P27 [74, 76–79]. Furthermore, HCC cell proliferation was found to decrease upon KDM5B knockdown due to transcriptional downregulation of E2F1 and E2F2 transcription factors [75].

KDM3A demethylase (also known as JMJD1A) was shown to be highly expressed in HCC samples compared to normal samples [80]. Moreover, its function was studied in hypoxic conditions, a main characteristic of tumours. The repression of KDM3A using the appropriate knockdown under hypoxic conditions inhibited HCC cell proliferation whereas its upregulation under the same conditions induced HCC cell growth due to a reduction of the demethylation of H3K9 at the promoter of the adrenomedullin gene and the consequent overexpression of this target [81, 82]. These results were validated in vivo [83]. Interestingly, this work showed that the silencing of KDM3A decreased phosphatidylinositol 3-kinase (PI3K) pathway activity which is a well-known oncogenic pathway and is often activated in hepatocellular carcinoma to promote survival and proliferation [84].

## Conclusion and further directions

It is clear from the examination of recent literature that dysregulation of histone methyltransferases and demethylases is a common phenotype in pre-cancerous liver disease and in full-blown HCC. The altered activity of these genes subsequently leads to changes in histone methyl marks at both global levels and more locally associated with specific genes. Since tumourigenesis involves hyperactivation of oncogenes and silencing of tumour suppressors, altered histone methylation could be implicated in the inappropriate transcriptional regulation of such genes.

**Fig. 4:**
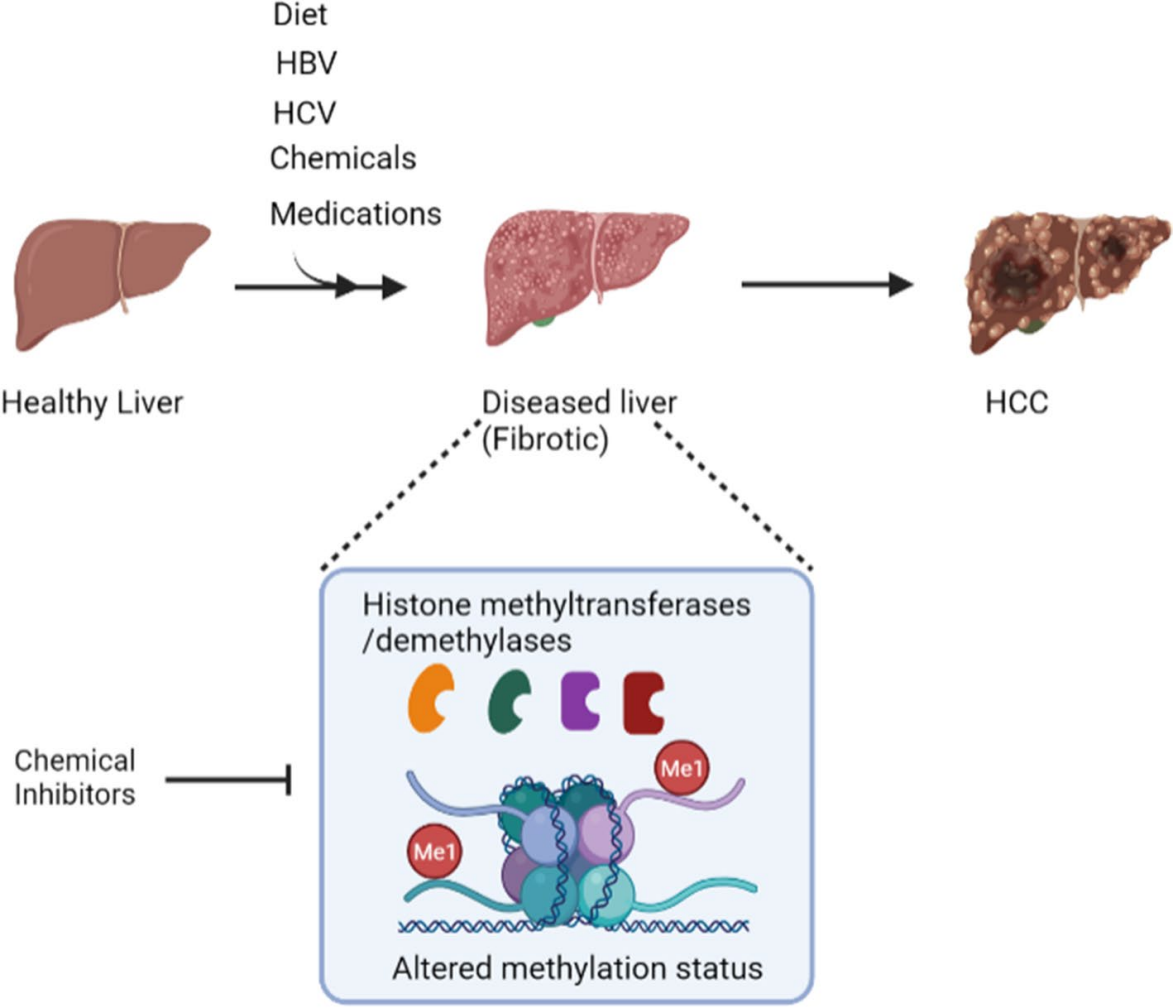
Histone methylation as a target for preventing progression of diseased liver to HCC. Exposure of healthy liver to agents such as unhealthy diet and viruses can cause chronic liver disease, with histone methylation implicated in this step. Targeting histone methylation in pre-cancerous disease liver, for example through the use of selective chemical inhibitors or medications, can potentially prevent or delay progression to HCC that is resistant to therapies

Of particular interest is the involvement of histone methyltransferases and demethylases in the establishment, maintenance and progression of pre-cancerous chronic liver diseases. As discussed, histone methylation has a role in the interaction between diet and NAFLD, chronic HBV infection through regulation of cccDNA transcription, and progression of HCV-infected livers to HCC even after effective antiviral treatments. The causal links from histone methylation to cell phenotypes are complex and are context- and cell-dependent. Nevertheless, targeting histone methylation in pre-cancerous chronic liver disease could offer new avenues for preventing the progression to highly lethal HCC (Fig. 4).

## Data Availability

This review article has no supporting data, and thus this section is not applicable.

## Abbreviations

cccDNA: Covalently closed circular DNA
CD36: Fatty acid translocase
ChIP-Seq: Chromatin immunoprecipitation sequencing
CIDEC: Fat-specific protein 27/cell death-inducing DFFA-like effector C
FABP4: Fatty acid binding protein 4
FGF21: Fibroblast growth factor-21
HBc: HB core protein
HBV: Hepatitis B virus
HBx: HBV X protein
HCC: Hepatocellular carcinoma
HCV: Hepatitis C virus
H3K27me3: Trimethylation of histone H3 lysine 27
H3K9: Histone H3 lysine 9
JmjC: α-Ketoglutarate-dependent Jumonji C domain
JMJD3: Jumonji D3 domain-containing protein
JMJ6: Jumonji domain-containing 6 protein
KMTs: Lysine methyltransferases
KMT1B: Suppressor of variegation 39 homolog 2
KMT2D: Mixed-lineage leukaemia 4 methyltransferase
lncRNA: Long noncoding RNA
LXRE: LXR response elements
LXRα: Ligand-activated liver X receptor α
MCD: Methionine/choline-deficient diet
NAFLD: Non-alcoholic fatty liver disease
NASH: Non-alcoholic steatohepatitis
NFkB: Nuclear factor kappa B
PADI4: Peptidyl arginine deiminase 4
PDK4: Pyruvate dehydrogenase kinase isoenzyme 4
PI3K: Phosphatidylinositol 3-kinase
PLIN2: Perilipin 2
PPARα: Nuclear receptor peroxisome proliferator activated receptor α
PPARγ2: Nuclear receptor peroxisome proliferator activated receptor γ isoform 2
PRMTs: Protein arginine methyltransferases
Sirt-1: Sirtuin 1
WDR5: WD repeat domain 5 protein
